# Senses of place: architectural design for the multisensory mind

**DOI:** 10.1186/s41235-020-00243-4

**Published:** 2020-09-18

**Authors:** Charles Spence

**Affiliations:** Department of Experimental Psychology, Crossmodal Research Laboratory, University of Oxford, Anna Watts Building, Oxford, OX2 6GG UK

**Keywords:** Multisensory perception, Architecture, The senses, Crossmodal correspondences

## Abstract

Traditionally, architectural practice has been dominated by the eye/sight. In recent decades, though, architects and designers have increasingly started to consider the other senses, namely sound, touch (including proprioception, kinesthesis, and the vestibular sense), smell, and on rare occasions, even taste in their work. As yet, there has been little recognition of the growing understanding of the *multisensory* nature of the human mind that has emerged from the field of cognitive neuroscience research. This review therefore provides a summary of the role of the human senses in architectural design practice, both when considered individually and, more importantly, when studied collectively. For it is only by recognizing the fundamentally multisensory nature of perception that one can really hope to explain a number of surprising crossmodal environmental or atmospheric interactions, such as between lighting colour and thermal comfort and between sound and the perceived safety of public space. At the same time, however, the contemporary focus on synaesthetic design needs to be reframed in terms of the crossmodal correspondences and multisensory integration, at least if the most is to be made of multisensory interactions and synergies that have been uncovered in recent years. Looking to the future, the hope is that architectural design practice will increasingly incorporate our growing understanding of the human senses, and how they influence one another. Such a multisensory approach will hopefully lead to the development of buildings and urban spaces that do a better job of promoting our social, cognitive, and emotional development, rather than hindering it, as has too often been the case previously.

## Significance statement

Architecture exerts a profound influence over our well-being, given that the majority of the world’s population living in urban areas spend something like 95% of their time indoors. However, the majority of architecture is designed for the eye of the beholder, and tends to neglect the non-visual senses of hearing, smell, touch, and even taste. This neglect may be partially to blame for a number of problems faced by many in society today including everything from sick-building syndrome (SBS) to seasonal affective disorder (SAD), not to mention the growing problem of noise pollution. However, in order to design buildings and environments that promote our health and well-being, it is necessary not only to consider the impact of the various senses on a building’s inhabitants, but also to be aware of the way in which sensory atmospheric/environmental cues interact. Multisensory perception research provides relevant insights concerning the rules governing sensory integration in the perception of objects and events. This review extends that approach to the understanding of how multisensory environments and atmospheres affect us, in part depending on how we cognitively interpret, and/or attribute, their sources. It is argued that the confusing notion of synaesthetic design should be replaced by an approach to multisensory congruency that is based on the emerging literature on crossmodal correspondences instead. Ultimately, the hope is that such a multisensory approach, in transitioning from the laboratory to the real world application domain of architectural design practice, will lead on to the development of buildings and urban spaces that do a better job of promoting our social, cognitive, and emotional development, rather than hindering it, as has too often been the case previously.

## Introduction

We are visually dominant creatures (Hutmacher, 2019; Levin, 1993; Posner, Nissen, & Klein, 1976). That is, we all mostly tend to think, reason, and imagine visually. As Finnish architect Pallasmaa (1996) noted almost a quarter of a century ago in his influential work *The eyes of the skin: Architecture and the senses,* architects have traditionally been no different in this regard, designing primarily for the eye of the beholder (Bille & Sørensen, 2018; Pallasmaa, 1996, 2011; Rybczynski, 2001; Williams, 1980). Elsewhere, Pallasmaa (1994, p. 29) writes that: “The architecture of our time is turning into the retinal art of the eye. Architecture at large has become an art of the printed image fixed by the hurried eye of the camera*.*” The famous Swiss architect Le Corbusier (1991, p. 83) went even further in terms of his unapologetically oculocentric outlook, writing that: “I exist in life only if I can see”, going on to state that: “I am and I remain an impenitent visual—everything is in the visual” and “one needs to see clearly in order to understand”. Commenting on the current situation, Canadian designer Bruce Mau put it thus: “We have allowed two of our sensory domains—sight and sound—to dominate our design imagination. In fact, when it comes to the culture of architecture and design, we create and produce almost exclusively for one sense—the visual.” (Mau, 2018, p. 20; see also Blesser & Salter, 2007).

Such visual dominance makes sense or, at the very least, can be explained or accounted for neuroscientifically (Hutmacher, 2019; Meijer, Veselič, Calafiore, & Noppeney, 2019). After all, it turns out that far more of our brains are given over to the processing of what we see than to dealing with the information from any of our other senses (Gallace, Ngo, Sulaitis, & Spence, 2012). For instance, according to Felleman and Van Essen (1991), more than half of the cortex is engaged in the processing of visual information (see also Eberhard, 2007, p. 49; Palmer, 1999, p. 24; though note that others believe that the figure is closer to one third). This figure compares to something like just 12% of the cortex primarily dedicated to touch, around 3% to hearing, and less than 1% given over to the processing of the chemical senses of smell and taste.^1^ Information theorists such as Zimmerman (1989) arrived at a similar hierarchy, albeit with a somewhat different weighting for each of the five main senses. In particular, Zimmermann estimated a channel capacity (in bits/s) of 10^7^ for vision, 10^6^ for touch, 10^5^ for hearing and olfaction, and 10^3^ for taste (gustation).

**Fig. 1:**
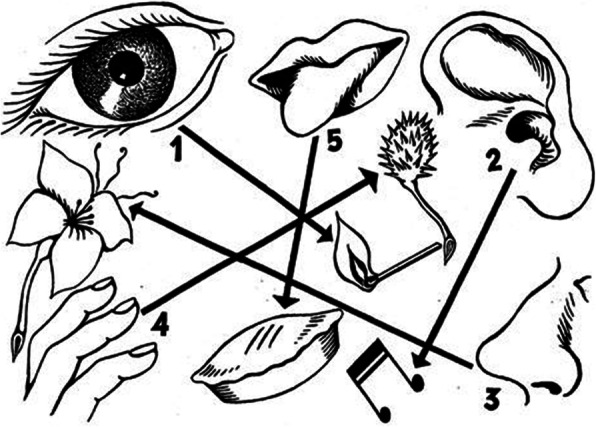
Heilig (1992) ranked the order in which he believed our attention to be captured by the various senses. According to Heilig’s rankings: vision, 70%; audition, 20%; olfaction, 5%; touch, 4%; and taste, 1%. Does the same hierarchy (and weighting) apply to our appreciation of architecture, one might wonder? And is attentional capture the most relevant metric anyway?

Figure 1 schematically illustrates the hierarchy of attentional capture by each of the senses as envisioned by Morton Heilig, the inventor of the *Sensorama,* the world’s first multisensory virtual reality apparatus (Heilig, 1962), when writing about the multisensory future of cinema in an article first published in 1955 (see Heilig, 1992). Nevertheless, while commentators from many different disciplines would seem to agree on vision’s current pre-eminence, one cannot help but wonder what has been lost as a result of the visual dominance that one sees wherever one looks in the world of architecture (“see” and “look” being especially apposite terms here).

While the hegemony of the visual (see Levin, 1993) is a phenomenon that appears across most aspects of our daily lives, the very ubiquity of this phenomenon certainly does not mean that the dominance of the visual should not be questioned (e.g., Dunn, 2017; Hutmacher, 2019). For, as Finnish architect and theoretician Pallasmaa (2011, p. 595) notes: “Spaces, places, and buildings are undoubtedly encountered as multisensory lived experiences. Instead of registering architecture merely as visual images, we scan our settings by the ears, skin, nose, and tongue.” Elsewhere, he writes that: “Architecture is the art of reconciliation between ourselves and the world, and this mediation takes place through the senses” (Pallasmaa, 1996, p. 50; see also Böhme, 2013). We will return later to question the visual dominance account, highlighting how our experience of space, as of anything else, is much more multisensory than most people realize.

### Review outline

While architectural practice has traditionally been dominated by the eye/sight, a growing number of architects and designers have, in recent decades, started to consider the role played by the other senses, namely sound, touch (including proprioception, kinesthesis, and the vestibular sense), smell, and, on rare occasions, even taste. It is, then, clearly important that we move beyond the merely visual (not to mention modular) focus in architecture that has been identified in the writings of Juhani Pallasmaa and others, to consider the contribution that is made by each of the other senses (e.g., Eberhard, 2007; Malnar & Vodvarka, 2004). Reviewing this literature constitutes the subject matter of the next section. However, beyond that, it is also crucial to consider the ways in which the senses interact too. As will be stressed later, to date there has been relatively little recognition of the growing understanding of the *multisensory* nature of the human mind that has emerged from the field of cognitive neuroscience research in recent decades (e.g., Calvert, Spence, & Stein, 2004; Stein, 2012).

The principal aim of this review is therefore to provide a summary of the role of the human senses in architectural design practice, both when considered individually and, more importantly, when the senses are studied collectively. For it is only by recognizing the fundamentally multisensory nature of perception that one can really hope to explain a number of surprising crossmodal environmental or atmospheric interactions, such as between lighting colour and thermal comfort (Spence, 2020a) or between sound and the perceived safety of public spaces (Sayin, Krishna, Ardelet, Decré, & Goudey, 2015), that have been reported in recent years.

At the same time, however, this review also highlights how the contemporary focus on synaesthetic design in architecture (see Pérez-Gómez, 2016) needs to be reframed in terms of the crossmodal correspondences (see Spence, 2011, for a review), at least if the most is to be made of multisensory interactions and synergies that affect us all. Later, I want to highlight how accounts of multisensory interactions in architecture in terms of synaesthesia tend to confuse matters, rather than to clarify them. Accounting for our growing understanding of crossmodal interactions (specifically the emerging field of crossmodal correspondences research) and multisensory integration will help to explain how it is that our senses conjointly contribute to delivering our multisensory (and not just visual) experience of space. One other important issue that will be discussed later is the role played by our awareness of the multisensory atmosphere of the indoor environments in which we spend so much of our time.

Looking to the future, the hope is that architectural design practice will increasingly incorporate our growing understanding of the human senses, and how they influence one another. Such a multisensory approach will hopefully lead to the development of buildings and urban spaces that do a better job of promoting our social, cognitive, and emotional development, rather than hindering it, as has too often been the case previously. Before going any further, though, it is worth highlighting a number of the negative outcomes for our well-being that have been linked to the sensory aspects of the environments in which we spend so much of our time.

### Negative health consequences of neglecting multisensory stimulation

It has been suggested that the rise in sick building syndrome (SBS) in recent decades (Love, 2018) can be put down to neglect of the olfactory aspect of the interior environments where city dwellers have been estimated to spend 95% of their lives (e.g., Ott & Roberts, 1998; Velux YouGov Report, 2018; Wargocki, 2001). Indeed, as of 2010, more people around the globe lived in cities than lived in rural areas (see UN-Habitat, 2010 and United Nations Department of Economic and Social Affairs, 2018). One might also be tempted to ask what responsibility, if any, architects bear for the high incidence of seasonal affective disorder (SAD) that has been documented in northern latitudes (Cox, 2017; Heerwagen, 1990; Rosenthal, 2019; Rosenthal et al., 1984). To give a sense of the problem of “light hunger” (as Heerwagen, 1990, refers to it), Terman (1989) claimed that as many as 2 million people in Manhattan alone experience seasonal affective and behavioural changes severe enough to require some form of additional light stimulation during the winter months.

According to Pallasmaa (1994, p. 34), Luis Barragán, the self-taught Mexican architect famed for his geometric use of bright colour (Gregory, 2016) felt that most contemporary houses would be more pleasant with only half their window surface. However, while such a suggestion might well be appropriate in Mexico, where Barragán’s work is to be found, many of us (especially those living in northern latitudes in the dark winter months) need as much natural light as we can obtain to maintain our psychological well-being. That said, Barragán is not alone in his appreciation of darkness and shadow. Some years ago, Japanese writer Junichirō Tanizaki also praised the aesthetic appeal of shadow and darkness in the native architecture of his home country in his extended essay on aesthetics, *In praise of shadows* (Tanizaki, 2001).

One of the problems with the extensive use of windows in northern climates is related to poor heat retention, an issue that is becoming all the more prominent in the era of sustainable design and global warming. One solution to this particular problem that has been put forward by a number of technology-minded researchers is simply to replace windows by the use of large screens that relay a view of nature for those who, for whatever reason, have to work in windowless offices (Kahn Jr. et al., 2008). However, the limited research that has been conducted on this topic to date suggests that the beneficial effects of being seated near to the window in an office building cannot easily be captured by seating workers next to such video-screens instead.

Similarly, the failure to fully consider the auditory aspects of architectural design may help to explain some part of the global health crisis associated with noise pollution interfering with our sleep, health, and well-being (Owen, 2019). The neglect of architecture’s fundamental role in helping to maintain our well-being is a central theme in Pérez-Gómez’s (2016) influential book *Attunement: Architectural meaning after the crisis of modern science.* Pérez-Gómez is the director of the History and Theory of Architecture Program at McGill University in Canada. Along similar lines, geographer J. Douglas Porteous had already noted some years earlier that: “Notwithstanding the holistic nature of environmental experience, few researchers have attempted to interpret it in a very holistic [or multisensory] manner.” (Porteous, 1990, p. 201). Finally, here, it is perhaps also worth noting that there are even some researchers who have wanted to make a connection between the global obesity crisis and the obesogenic environments that so many of us inhabit (Lieberman, 2006). The poor diet of multisensory stimulation that we experience living a primary indoor life has also been linked to the growing sleep crisis apparently facing so many people in society today (Walker, 2018).

## Designing for the modular mind

Researchers working in the field of environmental psychology have long stressed the impact that the sensory features of the built environment have on us (e.g., Mehrabian & Russell, 1974, for an influential early volume detailing this approach). Indeed, many years ago, the famous modernist Swiss architect Le Corbusier (1948) made the intriguing suggestion that architectural forms “work physiologically upon our senses.” Inspired by early work with the semantic differential technique, researchers would often attempt to assess the approach-avoidance, active-passive, and dominant-submissive qualities of a building or urban space. This approach was based on the pleasure, arousal, and dominance (PAD) model that has long been dominant in the field. However, it is important to stress that in much of their research, the environmental psychologists took a separate sense-by-sense approach (e.g., Zardini, 2005).

The majority of researchers have tended to focus their empirical investigations on studying the impact of changing the stimulation presented to just one sense at a time. More often than not, in fact, they would focus on a single sensory attribute, such as, for example, investigating the consequences of changing the colour (hue) of the lighting or walls (e.g., Bellizzi, et al., 1983; Bellizzi & Hite, 1992; Costa, Frumento, Nese, & Predieri, 2018; Crowley, 1993), or else just modulating the brightness of the ambient lighting (e.g., Gal, Wheeler, & Shiv, 2007; Xu & LaBroo, 2014). Such a unisensory (and, in some cases, unidimensional) approach undoubtedly makes sense inasmuch as it may help to simplify the problem of studying how design affects us (Malnar & Vodvarka, 2004). What is more, such an approach is also entirely in tune with the modular approach to mind that was so popular in the fields of psychology and cognitive neuroscience in the closing decades of the twentieth century (e.g., Barlow & Mollon, 1982; Fodor, 1983). At the same time, however, it can be argued that this sense-by-sense approach neglects the fundamentally multisensory nature of mind, and the many interactions that have been shown to take place between the senses.

The visually dominant approach to research in the field of environmental psychology also means that far less attention has been given over to studying the impact of the auditory (e.g., Blesser & Salter, 2007; Kang et al., 2016; Schafer, 1977; Southworth, 1969; Thompson, 1999), tactile, somatosensory or embodied (e.g., Heschong, 1979; Pallasmaa, 1996; Pérez-Gómez, 2016), or even the olfactory qualities of the built environment (e.g., Bucknell, 2018; Drobnick, 2002, 2005; Henshaw, McLean, Medway, Perkins, & Warnaby, 2018) than on the impact of the visual. Furthermore, until very recently, little consideration has been given by the environmental psychologists to the question of how the senses interact, one with another, in terms of their influence on an individual. This neglect is particularly striking given that the natural environment, the built environment, and the atmosphere of a space are nothing if not multisensory (e.g., Bille & Sørensen, 2018). In fact, it is no exaggeration to say that our response to the environments, in which we find ourselves, be they built or natural, is always going to be the result of the combined influence of all the senses that are being stimulated, no matter whether we are aware of their influence or not (this is a point to which we will return later).

Given that those of us living in urban environments, which as we have seen is now the majority of us, spend more than 95% of our lives indoors (Ott & Roberts, 1998), architects would therefore seem to bear at least some responsibility for ensuring that the multisensory attributes of the built environment work together to deliver an experience that positively stimulates the senses, and, by so doing, facilitates our well-being, rather than hinders it (see also Pérez-Gómez, 2016, on this theme). Crucially, however, a growing body of cognitive neuroscience research now demonstrates that while we are often unaware of, or at least pay little conscious attention to the subtle sensory cues that may be conveyed by a space (e.g., Forster & Spence, 2018), that certainly does not mean that they do not affect us. In fact, the sensory qualities or attributes of the environment have long been known to affect our health and well-being in environments as diverse as the hospital and the home, and from the office to the gym (e.g., Spence, 2002, 2003, 2021; Spence & Keller, 2019). What is more, according to the research that has been published to date, environmental multisensory stimulation can potentially affect us at the social, emotional, and cognitive levels.

It can be argued, therefore, that we all need to pay rather more attention to our senses and the way in which they are being stimulated than we do at present (see also Pérez-Gómez, 2016, on this theme). You can call it a mindful approach to the senses (Kabat-Zinn, 2005),^2^ though my preferred terminology, coined in an industry report published almost 20 years ago, is “sensism” (see Spence, 2002). Sensism provides a key to greater well-being by considering the senses holistically, as well as how they interact, and incorporating that understanding into our everyday lives. The approach also builds on the growing evidence of the nature effect (Williams, 2017) and the fact that we appear to benefit from, not to mention actually desire, the kinds of environments in which our species evolved. As support for the latter claim, consider only how it has recently emerged that most people set their central heating to a fairly uniform 17–23 °C, meaning that the average indoor temperature and humidity most closely matches the mild outdoor conditions of west central Kenya or the Ethiopian highlands (i.e., the place where human life is first thought to have evolved), better than anywhere else (Just, Nichols, & Dunn, 2019; Whipple, 2019).

## Architectural design for *each* of the senses

It is certainly not the case that architects have uniformly ignored the non-visual senses (e.g., see Howes, 2005, 2014; McLuhan, 1961; Pallasmaa, 1994, 2011; Ragavendira, 2017). For instance, in their 2004 book on *Sensory design*, Malnar and Vodvarka talk about challenging visual dominance in architectural design practice by giving a more equal weighting to all of the senses (Malnar & Vodvarka, 2004; see also Mau, 2019). Meanwhile, Howes (2014) writes of the sensory monotony of the bungalow-filled suburbs and of the corporeal experience of skyscrapers as their presence looms up before those on the sidewalk below. At the same time, however, there is also a sense in which it is the gaze of the inhabitants of those tall buildings who are offered the view that is prioritized over the other senses.

However, very often the approach as, in fact, evidenced by Malnar and Vodvarka (2004) has been to work one sense at a time. Until recently, that is, one finds exactly the same kind of sense-by-sense (or unisensory) approach in the worlds of interior design (Bailly Dunne & Sears, 1998), advertising (Lucas & Britt, 1950), marketing (Hultén, Broweus, & Dijk, 2009; Krishna, 2013; Lindstrom, 2005), and atmospherics (see Bille & Sørensen, 2018, on architectural atmospherics; and Kotler, 1974, on the theme of store atmospherics). Recently, there has been a growing recognition of the importance of the non-visual senses to various fields of design (Haverkamp, 2014; Lupton & Lipps, 2018; Malnar & Vodvarka, 2004). As yet, however, there has not been sufficient recognition of the extent to which the senses interact. As Williams (1980, p. 5) noted some 40 years ago: “Aside from meeting common standards of performance, architects do little creatively with acoustical, thermal, olfactory, and tactile sensory responses.” As we will see later, it is not clear that much has changed since.

### The look of architecture

**Fig. 2:**
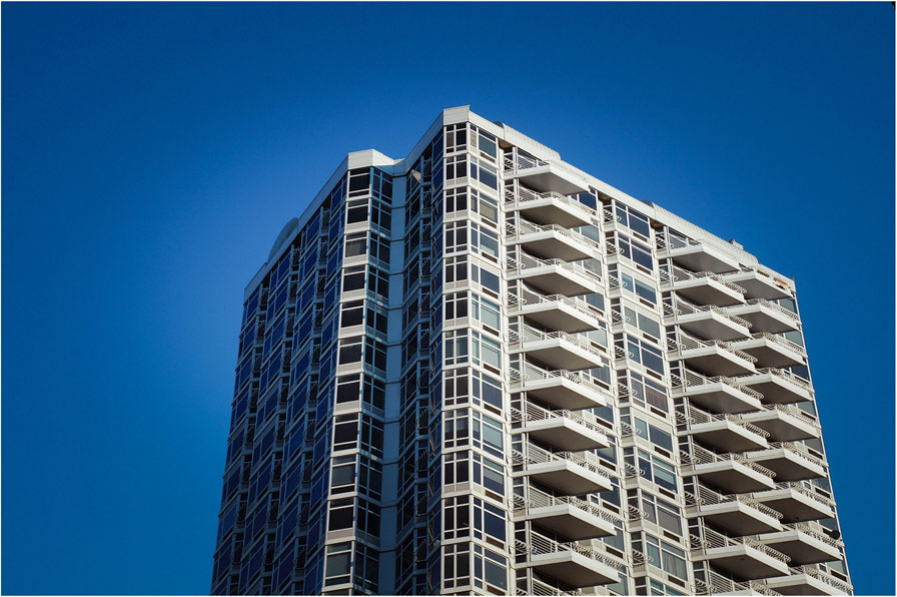
The Future apartment building at 200 East 32nd Street in Manhattan. Architectural design that appeals primarily to the eye? [Credit Jeffrey Zeldman, and reprinted under Creative Commons agreement]

There are a number of ways in which visual perception science can be linked to architectural design practice. For instance, think only of the tricks played on the eyes by the trapezoidal balconies on the famous *The Future* apartment building in Manhattan (see Fig. 2). They appear to slant downward when viewed from one side while appearing to slope upward instead, if viewed from the other. The causes of such a visual illusion can, at the very least, be meaningfully explained in terms of visual perception research (Bruno & Pavani, 2018).

**Fig. 3:**
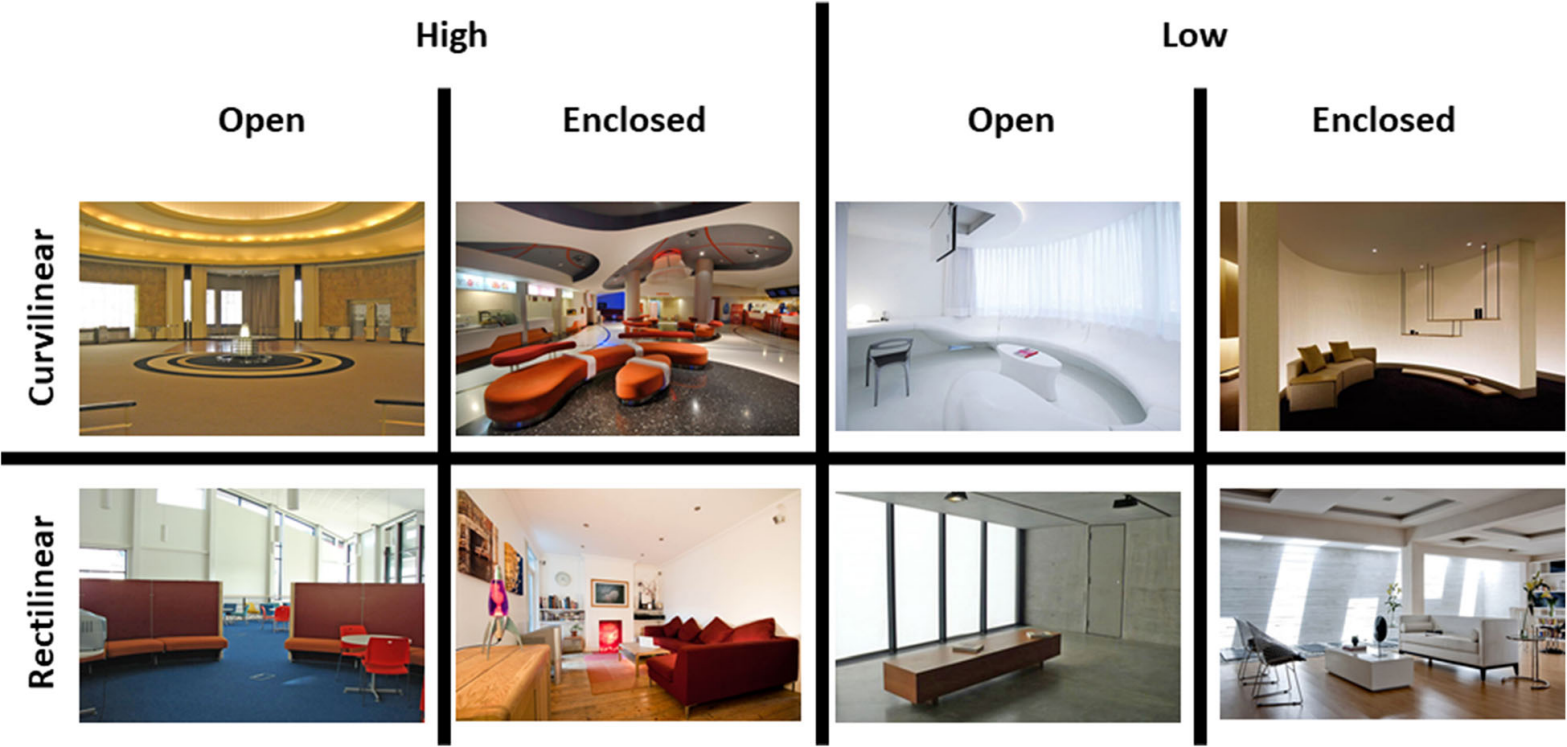
A selection of the interiors shown to participants in a neuroimaging study designed to assess viewers’ approach-avoidance motivation in response to curvilinear vs. rectilinear spaces. [High/Low roof; Open/Enclosed space.] [Figure reprinted with permission from Vartanian et al., 2013]

**Fig. 4:**
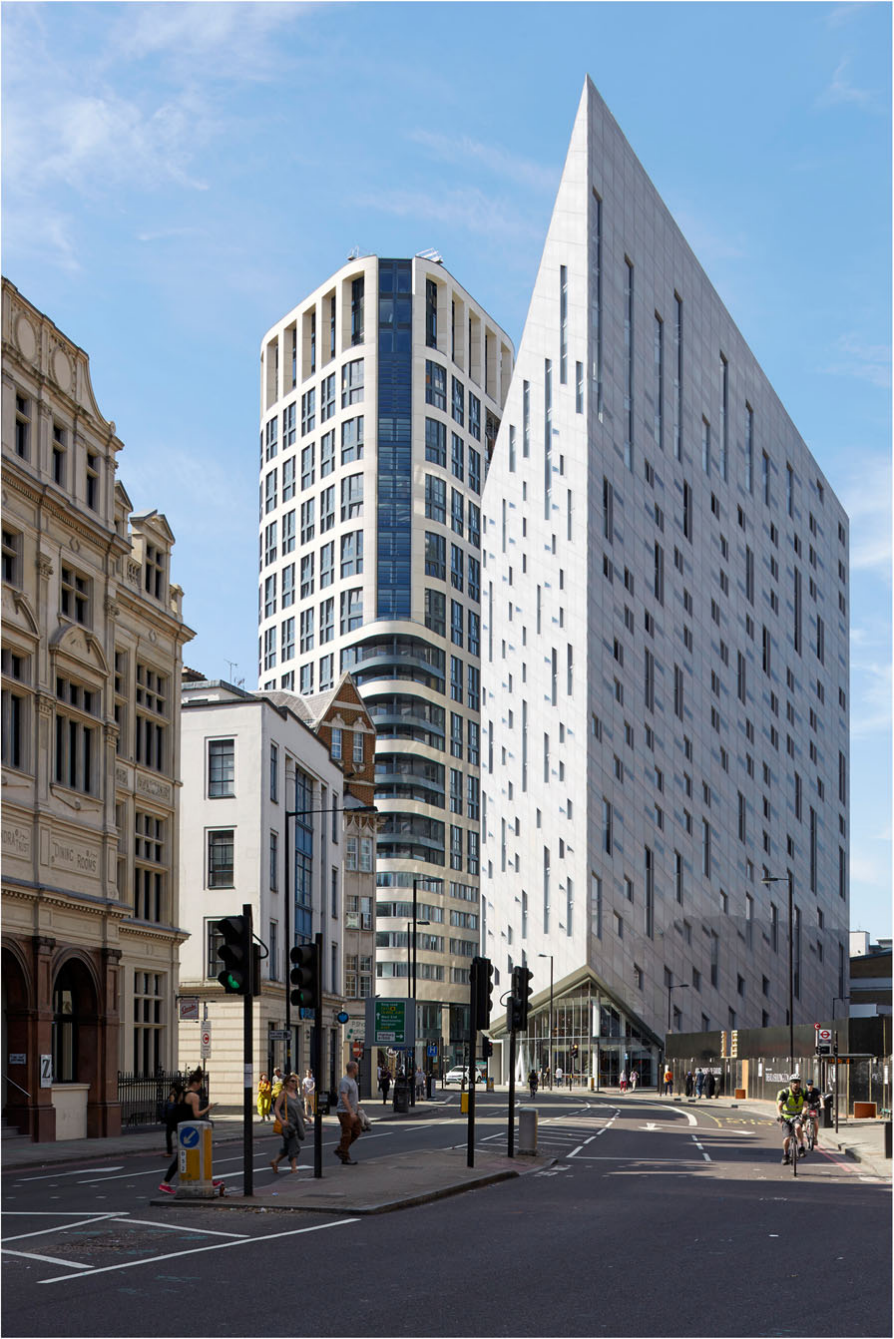
*Montcalm Shoreditch Signature Tower* Hotel, 151–157 City Road, London, completed 2015 by SMC Alsop Architects. What is lost when architectural design focuses on eye appeal? [Figure copyright Ian Ritchie, RA]

Cognitive neuroscientists have recently demonstrated that we have an innate preference for visual curvature, be it in internal space (Vartanian et al., 2013), or for the furniture that is found within that space (Dazkir & Read, 2012; see also Lee, 2018; Thömmes & Hübner, 2018). We typically rate curvilinear forms as being more approachable than rectilinear ones (see Fig. 3). Angular forms, especially when pointing downward/toward us, may well be perceived as threatening, and hence are somewhat more likely to trigger an avoidance response (Salgado-Montejo, Salgado, Alvarado, & Spence, 2017). As Ingrid Lee, former design director at IDEO New York put it in her book, *Joyful: The surprising power of ordinary things to create extraordinary happiness*: “Angular objects, even if they’re not directly in your path as you move through your home, have an unconscious effect on your emotions. They may look chic and sophisticated, but they inhibit our playful impulses. Round shapes do just the opposite. A circular or elliptical coffee table changes a living room from a space for sedate, restrained interaction to a lively center for conversation and impromptu games” (Lee, 2018, p. 142). One might consider here whether Lee’s comments can be scaled up to describe how we move through the city. Does the visually striking building shown in Fig. 4, for instance, really promote joyfulness and a carefree travel through the urban environment. It seems doubtful, given the evidence suggesting that viewing angular shapes, even briefly, has been shown to trigger a fear response in the amygdala, the part of the brain that is involved in emotion (e.g., LeDoux, 2003). Meanwhile, Liu, Bogicevic, and Mattila (2018) have noted how the round versus angular nature of the servicescape also influences the consumer response in service encounters.

The height of the ceiling has also been shown to exert an influence over our approach-avoidance responses, and perhaps even our style of thinking (Baird, Cassidy, & Kurr, 1978; Meyers-Levy & Zhu, 2007; Vartanian et al., 2015). However, here it should also be born in mind that the visual perception of space is significantly influenced by colour and lighting (Lam, 1992; Manav, Kutlu, & Küçükdoğu, 2010; Oberfeld, Hecht, & Gamer, 2010; von Castell, Hecht, & Oberfeld, 2018). Given many such psychological observations, it should perhaps come as no surprise to find that links between cognitive neuroscience and architecture have grown rapidly in recent years (Choo, Nasar, Nikrahei, & Walther, 2017; Eberhard, 2007; Mallgrave, 2011; Robinson & Pallasmaa, 2015). At the same time, however, it is also worth remembering that it has primarily been people’s response to examples or styles of architecture that have been presented visually (via a monitor), with the participant lying horizontal, that have been studied to date, given the confines of the brain-scanning environment (though see also Papale, Chiesi, Rampinini, Pietrini, & Ricciardi, 2016).^3^

At the same time, however, it is important to realize that it is not just our visual cortex that responds to architecture. For, as Frances Anderton writes in *The Architectural Review*: “We appreciate a place not just by its impact on our visual cortex but by the way in which it sounds, it feels and smells. Some of these sensual experiences elide, for instance our full understanding of wood is often achieved by a perception of its smell, its texture (which can be appreciated by both looking and feeling) and by the way in which it modulates the acoustics of the space.” (Anderton, 1991, p. 27). The multisensory appreciation of quality here linking to a growing body of research on multisensory *shitsukan* perception - *shitsukan*, the Japanese word for “a sense of material quality” or “material perception” (see Fujisaki, 2020; Komatsu & Goda, 2018; Spence, 2020b). The following sub-sections summarize some of the key findings on how the non-visual sensory attributes of the built and urban environment affect us, when considered individually.

### The sound of space: are you listening?

What a space sounds like is undoubtedly important (Bavister, Lawrence, & Gage, 2018; McLuhan, 1961; Porteous & Mastin, 1985; Thompson, 1999). Sounds can, after all, provide subtle cues as to the identity or proportions of a space, even hinting at its function (Blesser & Salter, 2007; Eberhard, 2007; Robart & Rosenblum, 2005). As Pallasmaa (1994, p. 31) notes: “Every building or space has its characteristic sound of intimacy or monumentality, rejection or invitation, hospitality or hostility.” However, more often than not, discussion around sound and architectural design tends to revolve around how best to avoid, or minimize, unwanted noise (see Owen, 2019, on growing concerns regarding the latter). Indeed, as J. Douglas Porteous notes: “with the rapid urbanization of the world’s population, far more attention is being given to noise than to environmental sound … Research has concentrated almost entirely upon a single aspect of sound, the concept of noise or ‘unwanted sound.’” (Porteous, 1990, p. 48). Some years earlier, Schafer (1977, p. 222) had made much the same point when he wrote that: “The modern architect is designing for the deaf …. The study of sound enters modern architecture schools only as sound reduction, isolation and absorption.” The fact that year-on-year, noise continues to be one of the top complaints from restaurant patrons, perhaps tells us all we need to know about how successful designers have been in this regard (see Spence, 2014, for a review; Wagner, 2018).

There is also an emerging story here regarding the deleterious effects of loud background noise, and the often-beneficial effects of music and soundscapes, on the recovery of patients in the hospital/healthcare setting (see Spence & Keller, 2019, for a review). Meanwhile, one of the main complaints from those office workers forced to move into one of the open plan offices that have become so popular (amongst employers, if not employees) in recent years (see ‘Redesigning the corporate office’, 2019) is around noise distraction (Borzykowski, 2017; Burkus, 2016; Evans & Johnson, 2000).^4^ Once again, one might want to ask what responsibility architects bear. Experimental evidence documenting the deleterious effect of open-plan working has been reported by a number of researchers (e.g., Bernstein & Turban, 2018; De Croon, Sluiter, Kuijer, & Frings-Dresen, 2005; Otterbring, Pareigis, Wästlund, Makrygiannis, & Lindström, 2018).

There is research ongoing in a number of countries to investigate the use of nature sounds, such as, for example, the sound of running water, to help mask other people’s distracting conversations (Hongisto, Varjo, Oliva, Haapakangas, & Benway, 2017). Intriguingly, however, it turns out that people’s beliefs about the source of masking sounds, especially in the case of ambiguous noise, can sometimes influence how much relief they provide (Haga, Halin, Holmgren, & Sörqvist, 2016). So, for instance, Haga and her colleagues played the same ambiguous pink noise with interspersed white noise to three groups of office-workers. To one control group, the experimenters said nothing, a second group of participants was told that they could hear industrial machinery noise, while a third group was told that they were listening to nature sounds, based on a waterfall, instead. Intriguingly, subjective restoration was significantly higher amongst those who thought that they were listening to the nature sounds than in those who thought that they were listening to industrial noise instead. As might have been expected, the results of the control group, fell somewhere in between.

**Fig. 5:**
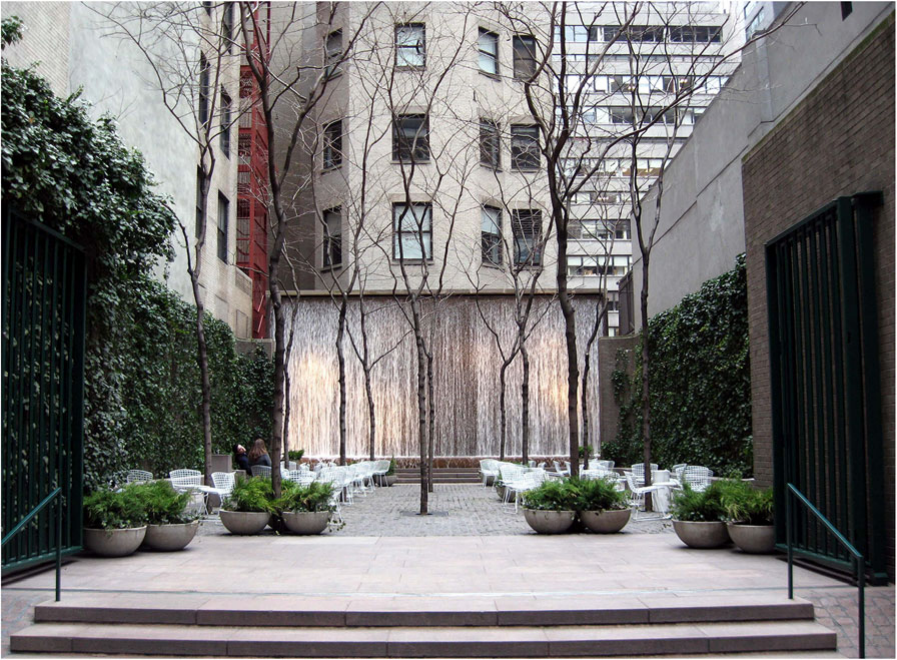
Paley Park, New York, by Zion and Breen in 1967. [Credit Jim Henderson, and reprinted under Creative Commons agreement]

Paley Park in New York has often been put forward as a particularly elegant solution to the problem of negating unwanted traffic noise in the context of urban design (e.g., Carroll, 1967; Prochnik, 2009). In 1967, the empty lot resulting from the demolition of the Stork Club on 53rd Street was transformed into a small public park (a so-called pocket park). The space was developed by Zion and Breen. In this case, the acoustic space, think only of the sounds, or better said noise, of the city, is effectively masked by the presence of a waterfall at the far end of the lot (see Fig. 5). What is more, the free-standing chairs allow the visitor to move closer to the waterfall should they feel the need to drown out a little more of the urban noise. The greenery growing thickly along the side walls also likely helps to absorb the noise of the city.

Music plays an important role in our experience of the built environment *-* think here only of the Muzak of decades gone by (Lanza, 2004). This is as true of the guest’s hotel experience (e.g., when entering the lobby) as it is elsewhere (e.g., in a shopping centre or bar, say).^5^ The sound that greets customers in the lobby is apparently very important to Ian Schrager, the Brooklyn-born entrepreneur who created fabled nightclub Studio 54 in New York. In recent years, he has been working with Marriott to launch The EDITION hotels in a number of major cities, including London and New York. Music plays a key role in the Schrager experience. As the entrepreneur puts it: “The sound of a hotel lobby is often dictated by monotonous, vapid lounge muzak – a zombie-like drone of new jazz and polite house, with the sole purpose of whiling away the waiting time between check-in and check-out.” As might have been expected, the music in the lobbies of The EDITION hotels is carefully curated (Eriksen, 2014, p. 27). However, the thumping noise of the music from the nightclub/bar that is often also an integral part of the experience offered by these hip venues means that meticulous architectural design is also required in order to limit the spread of unwanted noise through the rest of the building (e.g., so as not to disturb the sleep of those who may be resting in the rooms upstairs). Note here that there are also some increasingly sophisticated solutions - including sound-absorbing panels, as well as active noise cancellation systems - to dampen unwanted sound in open spaces such as restaurants and offices (Clynes, 2012).

### Designing for “the eyes of the skin”

The tactile element of architecture is often ignored. In fact, very often, the first point of physical contact with a building typically occurs when we enter or leave. Or, as Pallasmaa (1994, p. 33) once evocatively put it: “The door handle is the handshake of the building”. However, once inside a building, it is worth remembering that we will also typically make contact with flooring (Tonetto, Klanovicz, & Spence, 2014), hand rails (Spence, 2020d), elevator buttons, furniture, and the like (though this is, of course, likely to change somewhat in the era of pandemia). As Richard Sennett, author of *Flesh and Stone,* laments in his critical take on the sensory order of modernity: “sensory deprivation which seems to curse most modern buildings; the dullness, the monotony, and the tactile sterility which afflicts the urban environment” (Sennett, 1994, p. 15). The absence of tactile interest is also something that Witold Rybczynski author of *The Look of Architecture* acknowledges when writing that: “Although architecture is often defined in terms of abstractions such as space, light and volume, buildings are above all physical artifacts. The experience of architecture is palpable: the grain of wood, the veined surface of marble, the cold precision of steel, the textured pattern of brick.” (Rybczynski, 2001, p. 89). Notice here how Rybczynski mentions both texture and temperature, two of the key attributes of tactile sensation(see also Henderson, 1939). Temperature change, and change in the flooring material (tatami matting or cedarwood), is also something that the Tom museum for the blind in Tokyo also plays with deliberately (Classen, 1998, p. 150; Vorreiter, 1989; Wagner, 1989). There is also a braille poen on the knob of the exit door too.

The careful use of material can evoke tactility as the viewer (or occupant) imagines or mentally simulates what it would feel like to reach out and touch or caress an intriguing surface (Sigsworth, 2019; see also Lupton, 2002). Juhani Pallasmaa, who has perhaps written more than anyone else on the theme of the tactile, or haptic in architecture, writes that “Natural materials - stone, brick and wood - allow the gaze to penetrate their surfaces and they enable us to become convinced of the veracity of matter … But the materials of today - sheets of glass, enamelled metal and synthetic materials - present their unyielding surfaces to the eye without conveying anything of their material essence or age.” (Pallasmaa, 1994, p. 29).

Lisa Heschong, architect, and partner of architectural research firm Heschong Mahone Group, has written extensively on the theme of thermal (as opposed to textural) aspects of architectural design in her book *Thermal Delight in Architecture* (Heschong, 1979)*.* There, she points to examples such as the hearth, the sauna, and Roman and Japanese baths as archetypes of thermal delight about which rituals have developed, the shared experience reinforcing social bonds of affection and ceremony (see also Lupton, 2002; Papale et al., 2016). At this point, one might also want to mention the much-admired Therme Vals Spa by Peter Zumthor, in Switzerland with their use of different temperatures of both water and touchable surfaces (Ryan, 1997, though see also Mairs, 2017). The tactile element is, in other words, fundamental to the total (multisensory) experience of architectural design. This is true no matter whether the materiality is touched directly or not (i.e., merely seen, inferred, or imagined). So, for example, here one might only think about how looking at a cheap fake marble or wood veneer can make one feel, to realize that touch in often not required to assess material quality, or the lack thereof (see also Karana, 2010).

### An architecture of the chemical senses

Talking of an architecture of scent, or of taste (these two of the so-called chemical senses), might seem like a step too far. That said, one does come across titles such as *Eating Architecture* (Horwitz & Singley, 2004) and *An Architecture of Smell* (McCarthy, 1996; see also Barbara & Perliss, 2006).^6^ Unfortunately, however, all too often, consideration of the olfactory in architectural design practice has focused on the elimination of negative odours. When thinking about the mundane experience of odours in buildings, what immediately comes to mind includes the smell of wood (i.e., building materials), dust, mould, cleaning products, and flowers. As Eberhard (2007, p. 47) puts it: “We all have our favorite smells in a building, as well as ones that are considered noxious. A cedar closet in the bedroom is an easy example of a good smell. The terrible smell of a house that was ravaged by fire or floods is seared in the memory of those who have endured one of these disasters.” This is perhaps no coincidence, given that it tends to be the bad odours, rather than the neutral or positive ones, that have generally proved most effective in immersing us in an experience (Baus & Bouchard, 2017; see also Aggleton & Waskett, 1999). Research by Schifferstein, Talke, and Oudshoorn (2011) investigated whether the nightlife experience could be enhanced by the use of pleasant fragrance to mask the stale odour after the indoor smoking ban was introduced a few years ago. Once again, notice how the focus here is on the elimination of the negative stale odours rather than necessarily the introduction of the positive (the latter merely being introduced in order to mask the former).

Jim Drohnik captures the idea of olfactory absence when talking about not just the “white cube” mentality but the “anosmic cube” (Drobnick, 2005). The former phrase was famously coined by O’Doherty (1999, 2009) in order to describe the then-popular practice of displaying art in gallery spaces that were devoid of colour or any other form of visual distraction.^7^ Some years later, Jim Drobnik introduced the latter phrase in order to highlight the fact that too many spaces are seemingly deliberately designed to have no smell, nor to leave any lasting olfactory trace, either.^8^ And yet, at the same time, it is clear that odour of a space can be incredibly evocative too, as anecdotally noted by Pallasmaa (1994, p. 32) in the following quote: “The strongest memory of a space is often its odor; I cannot remember the appearance of the door to my grandfather’s farm-house from my early childhood, but I do remember the resistance of its weight, the patina of its wood surface scarred by a half century of use, and I recall especially the scent of home that hit my face as an invisible wall behind the door.” And thinking back to my memories of visiting my own grandfather, long since deceased, on his fairground wagon in Bradford, it was undoubtedly the intense smell of “derv” (English slang for diesel-engine road vehicle), the liquid diesel oil that was used for trucks at the time, that I can still remember better than anything else. The residents of buildings tend to adapt to the positive and neutral smells in the buildings we inhabit. This is evidenced by the fact that we are typically only aware of the smell of our own home, what some call building odour, or BO for short, when we return after a long trip away (Dalton & Wysocki, 1996; McCooey, 2008).

#### Sick building syndrome and the problem of poor olfactory design

Improving indoor air quality might well also provide an effective means of helping to alleviate some of the symptoms of sick building syndrome (SBS) that were mentioned earlier (Guieysse et al., 2008). It is certainly striking how many large outbreaks of this still-mysterious condition reported in the 1980s were linked to the presence of an unfamiliar smell in closed office buildings with little natural ventilation (Wargocki, Wyon, Baik, Clausen, & Fanger, 1999; Wargocki, Wyon, Sundell, Clausen, & Fanger, 2000). For instance, in June 1986, more that 12% of the workforce of 2500 people working at the Harry S. Truman State Office Building in Missouri came down with the symptoms of SBS over a 3-day period (Donnell Jr. et al., 1989). The symptoms presented by some of the workers (including dizziness and difficulty in breathing) were so severe they had to be rushed to the local hospital for emergency treatment. And while a thorough examination of the building subsequently failed to reveal the presence of any particular toxic airborne pollutants that might have been responsible for the outbreak, in the majority of cases, it turned out that the symptoms of SBS were preceded by the perception of unusual odours and inadequate airflow in the building.

According to Donnell Jr. et al. (1989), these complaints of odours may well have heightened the perception of poor air quality by some employees in the building. This, in turn, may have led to an epidemic anxiety state resulting in the SBS outbreak (Faust & Brilliant, 1981). In fact, workers suffering from SBS were more than twice as likely to have noticed a particular odour in the work area before the onset of their symptoms than those who were working in the same building who were unaffected by the outbreak.^9^ At the same time, however, it should also be borne in mind that our tendency to focus on what we see and hear means that we often exhibit olfactory anosmia to ambient scents (Forster & Spence, 2018).

To give a sense of the potential scale of the problem, Woods (1989) estimated that 30–70 million people in the USA alone are exposed to offices that manifest SBS. As such, anything (and everything) that can be done to reduce the symptoms associated with this reaction to the indoor environment (Finnegan, Pickering, & Burge, 1984) will likely have a beneficial effect on the health and well-being of many people. At the same time, however, it is perhaps also worth bearing in mind here that the incidence of SBS would seem to have declined in recent years (though see also Joshi, 2008; Magnavita, 2015; Redlich, Sparer, & Cullen, 1997), perhaps suggesting that building design/ventilation has improved as a result of the earlier outbreaks.^10^ That said, it is perhaps also worth noting that there continues to be some uncertainty as to whether the very real symptoms of SBS should be attributed to airborne pollutants, or may instead be better understood as a psychosomatic response to a particular environmental atmosphere (see Fletcher, 2005 and Love, 2018). What is more, there has been a move by some researchers to talk in terms of the less pejorative-sounding building-related symptoms (BRS) instead (Niemelä, Seppänen, Korhonen, & Reijula, 2006). One more psychological factor that may be relevant here concerns the feeling of a lack of control over one’s multisensory environment that many of those working in ventilated buildings where the windows cannot be opened manually have may indeed play a role in the elicitation of SBS.

#### Scent and the city: designing fragrant spaces

There are, however, signs that the situation is slowly starting to change with regards to the emphasis placed on olfaction in both architectural and urban design practice. For instance, a number of commentators have noted, not to mention sometimes been puzzled by, the distinctive, yet unexplained, pleasant - and hence, one assumes, deliberately introduced - fragrances that some new constructions appear to have. Just take the case of the Barclays Center arena in Brooklyn, NY, home of the Brooklyn Nets, as a case in point. On its opening in 2013, various commentators in the press drew attention to the distinctive, if not immediately identifiable, scent that appeared to pervade the space, and which appeared to have been added deliberately - almost as if it were intended to be a signature scent for the space (e.g., Albrecht, 2013; Doll, 2013; Martinez, 2013). That said, the idea of fragrancing public spaces dates back at least as far as 1913. In that year, at the opening of the Marmorhaus cinema in Berlin, the fragrance of Marguerite Carré, a perfume by Bourjois, Paris, was deliberately (and innovatively, at least for the time) wafted through the auditorium (Berg-Ganschow & Jacobsen, 1987). Meanwhile, in what may well be a sign of things to come, synaesthetic perfumer Dawn Goldsworthy and her scent design company 12:29 recently made the press after apparently creating a bespoke scent for a new US$40 million apartment in Miami (Schroeder, 2018). What further opportunities might there be to design distinctive “signature” scents for spaces/buildings, one might ask (Henshaw et al., 2018; Jones, 2006; Trivedi, 2006)?

Evidence that the olfactory element of design can be used to affect behaviour change positively includes, for example, the observation that people tend to engage in more cleaning behaviours when there is a hint of citrus in the air (De Lange, Debets, Ruitenburg, & Holland, 2012; Holland, Hendriks, & Aarts, 2005). In the future, it may not be too much of a stretch to imagine public spaces filled with aromatic flowers and blossoming trees, introduced with the aim of helping to discourage people from littering, and who knows, perhaps even reducing vandalism (see also Steinwald, Harding, & Piacentini, 2014). In terms of the cognitive mechanism underlying such crossmodal effects of scent on behaviour, the suggestion, at least in the citrus cleaning example just mentioned, is that smelling an ambient scent that we associate with clean and cleaning then activates, or primes, the associated concepts (Smeets & Dijksterhuis, 2014). Having been primed, the suggestion is thus that this makes it that bit more likely that we will engage in behaviours that are congruent or consistent with the primed concept (though see Doyen, Klein, Pichon, & Cleeremans, 2012).

Elsewhere, researchers have already demonstrated the beneficial effects that lavender, and other scents normally associated with aromatherapy, have on those who are exposed to them. So, for instance, the latter tend to show reduced stress, better sleep, and even enhanced recovery from illness (see Herz, 2009; Spence, 2003, for reviews; though see also Haehner, Maass, Croy, & Hummel, 2017). According to one commentator writing in The New York Times: “While these findings have obvious implications for health care, the opportunities for architecture and urban planning are particularly intriguing. Designers are trained to focus mostly on the visual, but the science of design could significantly expand designers’ sensory palette. Call it medicinal urbanism.” (Hosey, 2013). Effects on people’s mood resulting from exposure to ambient scent have been reported in some by no means all studies (Glass & Heuberger, 2016; Glass, Lingg, & Heuberger, 2014; Haehner et al., 2017; Weber & Heuberger, 2008). It remains somewhat uncertain though whether the beneficial effects of aromatherapy scents can be explained by priming effects, based on associative learning, as in the case of the clean citrus scents mentioned above (see Herz, 2009), versus via a more direct (i.e., less cognitively mediated) physiological route (cf. Harada, Kashiwadani, Kanmura, & Kuwaki, 2018).

**Fig. 6:**
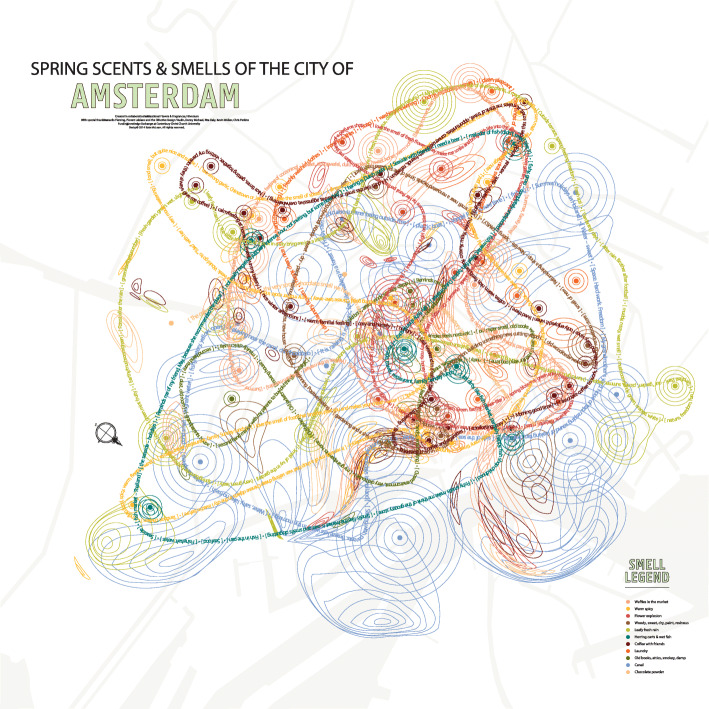
Scentscape of the city. Spring scents and smells of the city of Amsterdam by Kate McLean. [Credit “Spring Scents & Smells of the City of Amsterdam” © 2013-2014. Digital print. 2000 x 2000 mm. Courtesy of Kate McLean]

The olfactory scentscapes, and scent maps of cities, that have been discussed by various researchers (see Fig. 6) have also helped to draw people’s attention to the often rich olfactory landscapes offered by many urban spaces (e.g., https://sensorymaps.com/; Bucknell, 2018; Henshaw, 2014; Henshaw et al., 2018; Lipps, 2018; Lupton & Lipps, 2018; Margolies, 2006).

The notion of the healing garden has also seen something of a resurgence in recent years, and the benefits now, as historically, are likely to revolve, at least in part, around the healing, or restorative effect of the smell of flowers and plants (e.g., Pearson, 1991; see also Ottoson & Grahn, 2005). One building that is often mentioned in this regard, namely in terms of its olfactory design credentials, is the Silicon House by architects, SelgasCano, situated on the outskirts of Madrid (https://www.architectmagazine.com/project-gallery/silicon-house-6143). This house is set in what has been described as “a garden of smells”, which emphasize the olfactory, while also stressing the tactile elements of the design. Hence, while the olfactory aspects of architectural design practice have long been ignored, there are at least signs of a revival of interest in stimulating this sense through both architectural and urban design practice.

### Architectural taste

The British writer and artist Adrian Stokes once wrote of the “oral invitation of Veronese marble” (Stokes, 1978, p. 316). And while I must admit that I have never felt the urge to lick a brick, Pallasmaa (1996, p. 59) vividly recounts the urge that he once experienced to explore/connect with architecture using his tongue. He writes that: “Many years ago when visiting the DL James Residence in Carmel, California, designed by Charles and Henry Greene, I felt compelled to kneel and touch the delicately shining white marble threshold of the front door with my tongue. The sensuous materials and skilfully crafted details of Carlo Scarpa’s architecture as well as the sensuous colours of Luis Barragan’s houses frequently evoke oral experiences. Deliciously coloured surfaces of stucco lustro, a highly polished colour or wood surfaces also present themselves to the appreciation of the tongue.”

Perhaps aware of many readers’ presumed scepticism on the theme of the gustatory contribution to architecture,^11^ Pallasmaa writes elsewhere that: “The suggestions that the sense of taste would have a role in the appreciation of architecture may sound preposterous. However, polished and coloured stone as well as colours in general, and finely crafted wood details, for instance, often evoke an awareness of mouth and taste. Carlo Scarpa’s architectural details frequently evoke sensation of taste.” (Pallasmaa, 2011, p. 595). The suggestion here that “colours in general … often evoke … [a] taste” seemingly linking to the widespread literature on the crossmodal correspondences that have increasingly been documented between colour and basic tastes (see Spence et al., 2015, for a review). However, rather than describing this in terms of architecture that one can taste, one might more fruitfully refer to the growing literature on crossmodal correspondences instead (see below for more on this theme).

When, in his book *Architecture and the brain*, Eberhard (2007, p. 47) talks about what the sense of taste has to do with architecture, he suggests that: “You may not literally taste the materials in a building, but the design of a restaurant can have an impact on your ‘conditioned response’ to the taste of the food.” Environmental multisensory effects on tasting is undoubtedly an area that has grown markedly in interest in recent years (e.g., see Spence, 2020c, for a review). It is though worth noting that just as for the olfactory case, some atmospheric effects on tasting may be more cognitively-mediated (e.g., associated with the priming of notions of luxury/expense, or lack thereof) while others may be more direct, as when changing the colour (see Oberfeld, Hecht, Allendorf, & Wickelmaier, 2009; Spence, Velasco, & Knoeferle, 2014; Torrico et al., 2020) or brightness (Gal et al., 2007; Xu & LaBroo, 2014) of the ambient lighting changes taste/flavour perception.

### “An architecture of the seven senses”?

So far in this section, we have briefly reviewed the unisensory contributions of architectural design organized around each of the five main senses (vision audition, touch, smell, and taste). However, seemingly not content with the traditional five, Pallasmaa (1994) goes further in the title of one of his early articles entitled “An architecture of the seven senses.” While the text itself is not altogether clear, or explicit, on this point, the skeleton and muscles would appear to be the extra senses that Pallasmaa has in mind here. Indeed, the embodied response of people to architecture is definitely something that has captured the imagination, not to mention intrigued, a number of architectural theorists in recent years (e.g., see Bloomer & Moore, 1977; Pallasmaa, 2011; Pérez-Gómez, 2016).

**Fig. 7:**
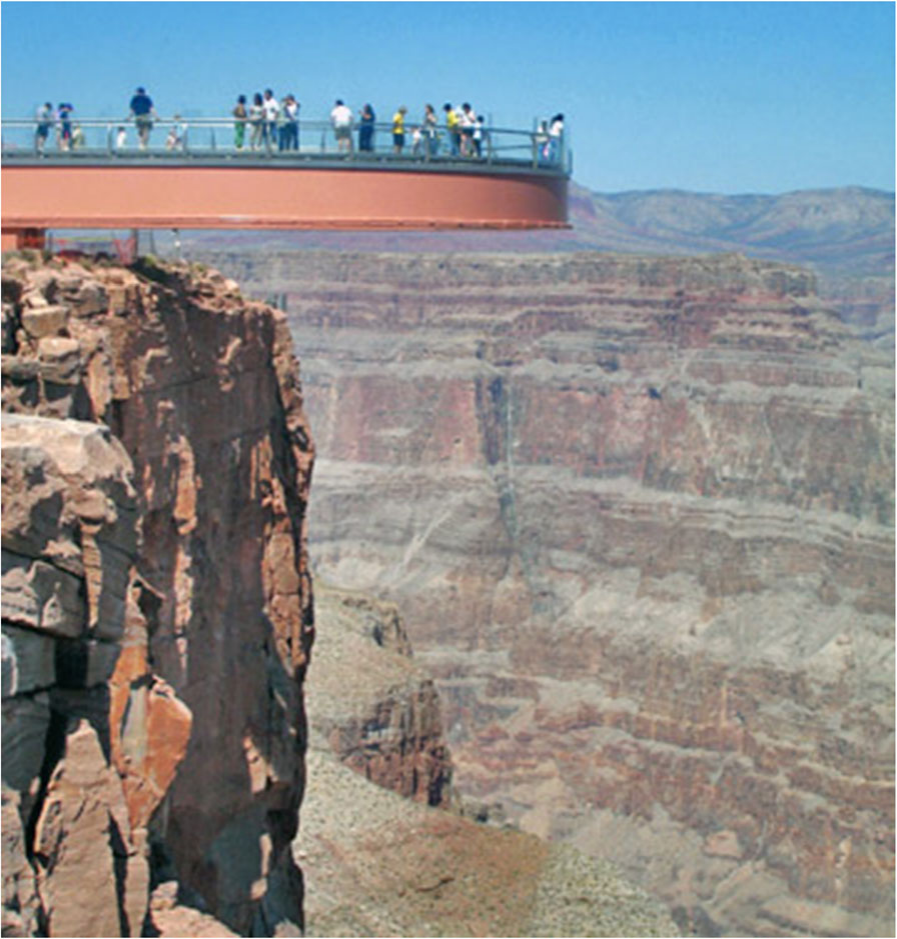
Skywalk from outside ledge. [Attribution: Complexsimplellc at English Wikipedia reprinted under Creative Commons agreement]

The vestibular sense is also worthy of mention here (see Gulden & Grüsser, 1998; Indovina et al., 2005). Anyone who has tried out one of the VR simulations of walking along the outside ledge of a tall building will have had the feeling of vertigo. Normally, architects presumably avoid designing structures that may give rise to such discombobulating feelings. That said, the recent increase in popularity of transparent viewing platforms, and bridges, shows that, on occasion, architects are not beyond emphasizing the important contribution made by this normally “silent” sense. For instance, The Grand Canyon Skywalk is a horseshoe-shaped cantilever bridge with a glass walkway at Eagle Point, Arizona that allows visitors to stand 500–800 ft. (150–240 m) above the canyon floor (Yost, 2007). Opened in 2007, by 2015, it had attracted more than a million visitors (see Fig. 7). While popular, it is perhaps worth noting that a number of such attractions have recently been closed down in parts of China due to safety fears (Ellis-Petersen, 2019). Walking on such structures likely also make people more aware of their own corporeality too, thus engaging the proprioceptive and kinaesthetic senses too. On a more mundane level, Heschong (1979, p. 34) draws attention to the importance of bodily movement in the case of the porch swing whose self-propelled movement, prior to air-conditioning, would have been a thermal necessity in the summer months in the southern states of the USA.

Consideration of the putatively embodied response to architecture might lead one back to Hall’s (1966) seminal early notion of “proxemics”. Hall used the latter term to describe the differing response to stimuli as a function of their distance from the viewer’s body. It is certainly easy to imagine this linking to contemporary notions concerning the different regions of personal space that have been documented around an observer (e.g., Previc, 1998; Spence, Lee, & Stoep, 2017). However, while these terms might sound more or less synonymous to cognitive neuroscientists, Malnar and Vodvarka (2004), both licensed architects, choose to take a much more cautious stance concerning these terms, treating them as referencing distinct phenomena in their own book on sensory design.

### Interim summary

While the impact of each of the senses, however many there might be, can undoubtedly be analysed in isolation, as has largely been attempted in the preceding sections, the fact of the matter is that they interact one with another in terms of determining our response to the environment, be it built or natural. So, having briefly addressed the contribution of each of the senses to architectural design practice, when studied individually, the next question to consider is how the senses interact in the perception of environment/atmosphere, as they do in many other aspects of our everyday perception. After all, as Malnar notes: “The point of immersing people within an environment is to activate the full range of the senses.” (Malnar, 2017, p. 146). Pallasmaa (2000, p. 78) makes a similar point writing that: “Every significant experience of architecture is multi-sensory; qualities of matter, space and scale are measured by the eye, ear, nose, skin, tongue, skeleton and muscle.” (cf. Rasmussen, 1993).

Malnar and Vodvarka (2004, p. ix) set the scene for the discussion with the opening lines of the preface of their book on sensory design in architecture, where they write: “What if we designed for all our senses? Suppose, for a moment, that sound, touch, and odour were treated as the equals of sight, and that emotion was as important as cognition. What would our built environment be like is sensory response, sentiment, and memory were critical design factors, more vital even than structure and program?” Indeed, those who take up the challenge of designing for the multisensory mind might well take a tip from one commentator, writing in *Advertising Age* when talking about product innovation who suggested that: “… the most successful new products appeal on both rational and emotional levels to as many senses as possible.” (Neff, 2000, p. 22). Architectural design practice, I suggest, would be well-advised to strive for much the same in order to optimally stimulate the multisensory mind.

Although not the primary interest of the present review, it is perhaps also worth noting in passing, how a very similar debate on the importance of designing for the non-visual senses has been playing out amongst those interested specifically in landscape design/architecture (Lynch & Hack, 1984; Mahvash, 2007; Treib, 1995). The garden is a multisensory space and as Mark Treib wrote once in an essay entitled “Must landscape mean?”: “Today might be a good time to once more examine the garden in relation to the senses.”

## Designing for the multisensory mind: architectural design for *all* the senses

The architect must act as a composer that orchestrates space into a synchronization for function and beauty through the senses – and how the human body engages space is of prime importance. As the human body moves, sees, smells, touches, hears and even tastes within a space – the architecture comes to life.

The rhythm of an architecture can be felt by occupants as a result of the architect’s composition – or arrangement of all the sensorial qualities of space. By arranging spatial sensorial features, an architect can lead occupants through the functional and aesthetic rhythms of a created place. Architectural building for all the senses can serve to move occupants – elevating their experience. (quote from a blogpost by Lehman, 2009).

One of the most exciting developments in cognitive neuroscience in recent decades has been the growing realization that perception/experience is far more multisensory than anyone had realized (e.g., Bruno & Pavani, 2018; Calvert et al., 2004; Levent & Pascual-Leone, 2014; Stein, 2012). That is, what we hear and smell, and what we think about the experience, is often influenced by what we see, and vice versa (Calvert et al., 2004; Stein, 2012). The senses talk to, and hence influence, one another all the time, though we often remain unaware of these cross-sensory interactions and influences. In fact, wherever neuroscientists look in the human brain, activity appears to be modulated by what is going on in more than one sense, leading, increasingly, to talk of the multisensory mind (Ghazanfar & Schroeder, 2006; Talsma, 2015). The key question here must therefore be what implications this growing realization of the ubiquity of multisensory cross-talk has for the field of architectural design practice?

The problem is that, as yet, there has been relatively little research directed at the question of how atmospheric/environmental multisensory cues actually interact. Mattila and Wirtz (2001, pp. 273–274) drew attention to this lacuna some years ago when writing that: “Past studies have examined the effects of individual pleasant stimuli such as music, color or scent on consumer behavior, but have failed to examine how these stimuli might interact.” At the outset, when starting to consider the multisensory perception of architecture, it is worth noting that it is rarely something that we attend to. Indeed, as Benjamin (1968, p. 239) once noted: “Architecture has always represented the prototype of a work of art the reception of which is consummated in a state of distraction.” To the extent that such a view is correct, one can say that multisensory architecture is rarely foregrounded in our attention/experience. Juhani Pallasma, meanwhile, has suggested that: “An architectural experience silences all external noise; it focuses attention on one’s very existence.” (Pallasmaa, 1994, p. 31). Once again, the suggestion here would appear to be that attention is directed away from the building and toward the individual and their place in the world. Given that, on an everyday basis, architecture is typically not foregrounded in our attention/experience, one might legitimately wonder as to whether the multisensory integration of atmospheric/environmental cues takes place, given that they are so often unattended.

According to the laboratory research that has been published on this question to date, the evidence would appear to suggest that while the multisensory integration of unattended cues relating to an object or event certainly can occur, it is by no means guaranteed to do so (see Spence & Frings, 2020, for a review). Perhaps the more fundamental question here, though, is whether we need to attend to ambient/environmental sensory cues for them to influence us. However, the research that has been published to date would appear to suggest that very often environmental cues influence us even when we are not consciously aware of, or thinking about them.

One particularly striking example of this was reported by researchers who manipulated whether French or German music was played in a supermarket (North, et al., 1997, 1999). The results showed that the majority of the wine purchased was French when French music was played, with this reversing to a majority of German wines being sold when German music was played. The even more striking aspect of these results was the fact that the majority of those interviewed after coming away from the tills denied that the background music had any influence over the choices they made. A number of studies have also shown that scents that we are unaware of, either because they are presented just below the perceptual threshold or because we have become functionally anosmic to their constant presence, can nevertheless still influence us (Li, Moallem, Paller, & Gottfried, 2007). Similarly, there is also a suggestion that inaudible infrasound waves (i.e., < 20 Hz) may also affect people without their necessarily being aware of their presence (Weichenberger et al., 2017). Meanwhile, in terms of visual annoyance, it has been reported that flickering LED lights that look no different to the naked eye can nevertheless trigger a significantly greater number of headaches that non-flickering lights (e.g., see Wilkins, 2017; Wilkins, Nimmo-Smith, Slater, & Bedocs, 1989). Once again, therefore, this suggests that ambient sensory phenomena do not necessarily need to be perceptible in order to affect us, adversely or otherwise.

### On the benefits of multisensory design: bringing it all together

**Fig. 8:**
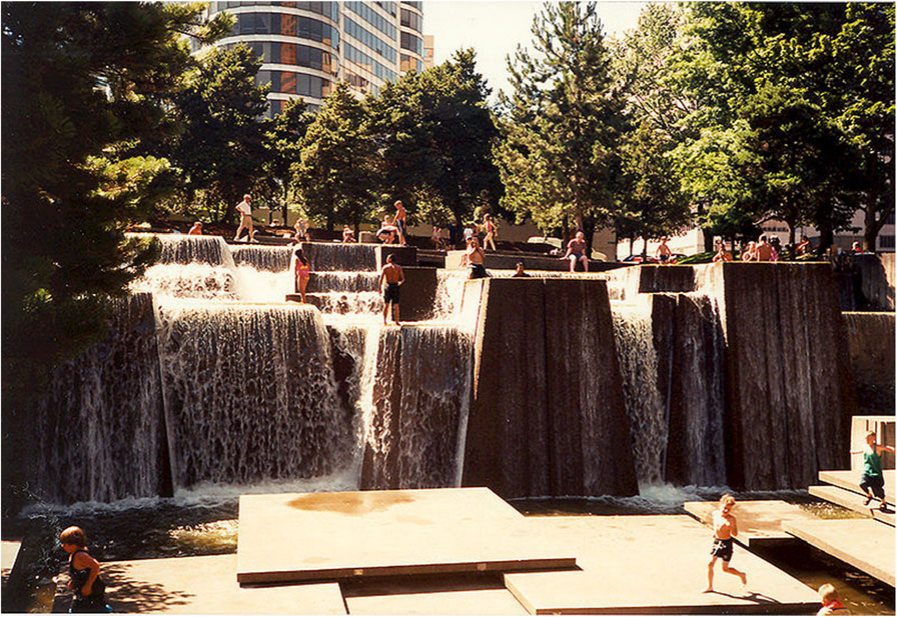
The Ira Keller Fountain, Portland Oregon. According to Pallasmaa (2011), p. 596) this is “An architecture for all the senses including the kinaesthetic and olfactory senses.” Once again, the auditory element is provided by the sound of falling water

One demonstration of just how dramatic the benefits of designing for multiple senses can be was reported by Kroner, Stark-Martin, and Willemain (1992) in a technical report. These researchers examined the effects of an office make-over when a company moved to a new office building. The employees in the new office were given individual control of the temperature, lighting, air quality, and acoustic conditions where they were working. Productivity increased by approximately 15% in the new building. When the individual control of the ambient multisensory environment was disabled in the new building, performance fell by around 2% instead. Trying to balance the influence of each of the senses is one of the aims of Finnish architect Juhani Pallasmaa, whose name we have come across at several points already in this text. As Steven Holl notes in the preface to Pallasmaa’s *The eyes of the skin*: “I have experienced the architecture of Juhani Pallasmaa, … The way spaces feel, the sound and smell of these places, has equal weight to the way things look.” (Pallasmaa, 1996, p. 7). One example of multisensory architectural design to which Juhani Pallasmaa draws attention in several of his writings is the Ira Keller Fountain, Portland Oregon (see Fig. 8).

### On the multisensory integration of atmospheric/environmental cues

To date, only a relatively small number of studies have directly studied the influence of combined ambient/atmospheric cues on people’s perception, feelings, and/or behaviour. Mattila and Wirtz (2001) conducted one of the first sensory marketing studies to be published in this area. These researchers manipulated the olfactory environment (no scent, a low-arousal scent (lavender), or a high-arousal scent (grapefruit)) while simultaneously manipulating the presence of music (no music, low-arousal music, or high-arousal music). When the scent and music were congruent in terms of their arousal potential, the customers rated the store environment more positively, exhibited higher levels of approach and impulse-buying behaviour, and expressed more satisfaction. There is, though, always a very real danger of sensory overload if the combined multisensory input becomes too stimulating (see Malhotra, 1984; Simmel, 1995).

Meanwhile, in another representative field study, Sayin et al. (2015) investigated the impact of presenting ambient soundscapes in an underground car park in Paris. In particular, they assessed the effects of introducing western European birdsong or classical instrumental music by Albinoni to the three normally silent stairwells used by members of the general public when exiting the car park. A total of 77 drivers were asked about their feelings on their way out. Birdsong was found to work best in terms of enhancing the perceived safety of the situation - in this case by around 6%. This despite the fact that all of those who were quizzed realized that the sounds that they had heard were coming from loudspeakers.^12^ In an accompanying series of laboratory studies, Sayin et al.’s participants were shown a 60-s first-person perspective video that had been taken in the same Paris car park, or else a short video of someone walking through a metro station in Istanbul. Once again, participants were asked about how safe it felt, about perceived social presence, and about their willingness to purchase a monthly metro pass. Even under these somewhat contrived experimental conditions, the presence of an ambient soundscape once again increased perceived safety as well as the participants’ self-reported intention to purchase a season ticket. It was, though, the sound of people singing Alleluia that proved most effective in terms of enhancing perceived safety amongst those watching the videos.^13^ It is, however, worth bearing in mind here that many of the key results reported in this study were only borderline significant. As such, adequately-powered replication would be a good idea before too much weight is given to these intriguing findings.

Recently, Ba and Kang (2019) documented crossmodal interactions between ambient sound and smell in a laboratory study that was designed to capture the sensory cues that might be encountered in a typical urban environment. These researchers decided to pair the sounds of birds, conversation, and traffic, with the smells of flowers (lilac, osmanthus), coffee, or bread, at one of three levels (low, medium, or high) in each modality. A complex array of interactions was observed, with increasing stimulus intensity sometimes enhancing the participants’ comfort ratings, while sometimes leading to a negative response instead. While Ba and Kang’s results defy any simple synopsis, given the complex pattern of results reported, their findings nevertheless clearly suggest that sound and scent interact in terms of influencing people’s evaluation of urban design.

The colour of the ambient lighting in an indoor environment has also been shown to influence the perceived ambient temperature and thermal comfort of an environment (e.g., Candas & Dufour, 2005; Tsushima, et al., 2020; Winzen, Albers, & Marggraf-Micheel, 2014). For instance, in one representative study, Winzen and colleagues reported that illuminating a simulated aircraft cabin in warm yellow vs. cool blue-coloured lighting exerted a significant influence over people’s self-reported thermal comfort. The participants rated the environment as feeling significantly warmer under the warm (as compared to the cool) lighting colour. One can only really make sense of such findings from a multisensory perspective (see Spence, 2020a, for a review).

Taken together, then, the results of the representative selection of studies reported in this section demonstrate that our perception of, and/or response to, multisensory environments are undoubtedly influenced by the combined influence of environmental/atmospheric cues in different sensory modalities. So, in contrast to the quote from Mattila and Wirtz (2001) that we came across a few pages ago, there is now a growing body of empirical research out there demonstrating that atmospheric cues presented in different sensory modalities, such as music, scents, and visual stimuli combine to influence how alerting, or pleasant, a particular environment, or stimulus (such as, for example, a work of art), is rated as being (e.g., Banks, Ng, & Jones-Gotman, 2012; Battacharya & Lindsen, 2016).

### Sensory congruency

In their book, *Spaces speak, are you listening*?, Blesser and Salter draw the reader’s attention to the importance of audiovisual congruency in architectural design. They write that: “Aural architecture, with its own beauty, aesthetics, and symbolism, parallels visual architecture. Visual and aural meanings often align and reinforce each other. For example, the visual vastness of a cathedral communicates through the eyes, while its enveloping reverberation communicates through the ears.” (Blesser & Salter, 2007, p. 3). However, they also draw attention to the incongruency that one experiences sometimes: “Although we expect the visual and aural experience of a space to be mutually supportive, this is not always the case. Consider dining at an expensive restaurant whose decorations evoke a sense of relaxed and pampered elegance, but whose reverberating clatter produces stress, anxiety, isolation, and psychological tension, undermining the possibility of easy social exchange. The visual and aural attributes produce a conflicting response.” (Blesser & Salter, 2007, p. 3).

**Fig. 9:**
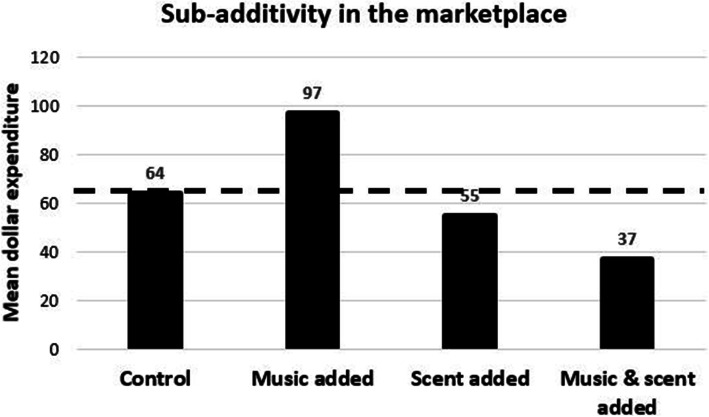
Morrin and Chebat (2005). Sales figures (unplanned purchases) in mall as a function of music, scent, or the combination of the two. In this case, multisensory stimulation led to a significant reduction in sales, perhaps because low-tempo music was combined with a likely-alerting citrus scent

Regardless of whether atmospheric/environmental sensory cues are integrated or not, one general principle underpinning our response to multisensory combinations of environmental cues is that those combinations of stimuli that are “congruent” (whatever that term means in this context) will tend to be processed more fluently, and hence be liked more, than those combinations that are deemed incongruent, and hence will often prove more difficult, and effortful, to process (Reber, 2012; Reber, Schwarz, & Winkielman, 2004; Reber, Winkielman, & Schwartz, 1998; Winkielman, Schwarz, Fazendeiro, & Reber, 2003; Winkielman, Ziembowicz, & Nowak, 2015).^14^ Indeed, it was the putative sensory incongruency between a relaxing slow-tempo music and arousing citrus scent that was put forward as a possible explanation for why Morrin and Chebat (2005) found that adding scent and sound in the setting of the shopping mall reduced unplanned purchases as compared to either of the unisensory interventions amongst almost 800 shoppers in one North American Mall (see Fig. 9).

Congruency can, of course, be defined at multiple levels. For instance, as we have seen already in this section, sensory cues may be more or less congruent in terms of their arousal/relaxation potential (e.g., Homburg, Imschloss, & Kühnl, 2012; Mattila & Wirtz, 2001). Mahvash (2007, pp. 56–57) talks about the use of congruent cues to convey the notion of coolness: “… the Persian garden with its patterns of light and shadow, reflecting pools, gurgling fountains, scents of flowers and fruits, and gentle cool breezes 'offers an amazing richness of variety of sensory experiences which all serve to reinforce the pervasive sense of coolness'.” However, different sensory inputs may also be deemed congruent or not in terms of their artistic style (see Hasenfus, Martindale, & Birnbaum, 1983; Muecke & Zach, 2007; cf. Hersey, 2000, pp. 37–41). It was stylistic congruency that was manipulated in a couple of experiments, conducted both online and in the laboratory by Siefkes and Arielli (2015). These researchers had their participants explicitly concentrate on and evaluate the style of the buildings shown in one of two architectural styles (baroque or modern - a short video showing five baroque buildings; there were also a short video, focusing on five modern buildings instead). Their results revealed that the buildings were rated as looking more balanced, more coherent, and to a certain degree, more complete,^15^ when viewed while listening to music that was congruent (e.g., baroque architecture with baroque music - specifically Georg Philipp Telemann’s, Concerto Grosso in D major, TWV 54:D3 (1716)) rather than incongruent (e.g., baroque architecture with Philip Glass track from the soundtrack to the movie Koyaanisqatsi).

Before moving on, though, it is worth noting that in this study, as in many of the other studies reported in this section, there is a possibility that the design of the experiments themselves may have resulted in the participants concerned paying rather more attention to the atmospheric/environmental cues (and possibly also their congruency) than is normally likely to be the case when, as was mentioned earlier, the architecture itself fades into the background. Ecological validity may, in other words, have been compromised to a certain degree.

One of the other examples of incongruency that one often comes across is linked to the growing interest in biophilic design. As Pallasmaa (1996, p. 41) notes: “A walk through a forest is invigorating and healing due to the constant interaction of all sense modalities; Bachelard speaks of ‘the polyphony of the senses’. The eye collaborates with the body and the other senses. One’s sense of reality is strengthened and articulated by this constant interaction. Architecture is essentially an extension of nature into the man-made realm …”^16^ No wonder, then, that many designers have been exploring the benefits of bringing elements of nature into interior spaces in order to boost the occupants’ mood and aid relaxation (Spence, 2021). However, one has to ask whether the benefits of adding the sounds of a tropical rainforest to a space such as the shopping area of Glasgow airport, say (Treasure, 2007), really outweigh the cognitive dissonance likely elicited by hearing such sounds in such an incongruous setting? Similarly, a jungle soundscape was incorporated into the children’s section of Harrods London Department store a few years ago (Harrods’ Toy Kingdom - The Sound Agency | Sound Branding” https://www.youtube.com/watch?v=EVUUG6VvFKQ). Nature soundscapes have also been introduced into Audi car salesrooms, not to mention BP petrol station toilet facilities (Bashford, 2010; Treasure, 2007). It is worth noting here that given the important role that congruency has been shown to play at the level of multisensory object/event perception, there is currently a stark paucity of research that has systematically investigated the relevance/importance of congruency at the level of multisensory ambient, or environmental, cues. As the quotes earlier in this section make clear, it is something to which some architects are undoubtedly sensitive, and on which they already have an opinion. Yet the relevant underpinning research still needs to be conducted.

Ultimately, therefore, while the congruency of atmospheric/environmental cues can be defined in various ways, and while incongruency is normally negatively valenced (because it is hard to process),^17^ issues of (in)congruency may often simply not be an issue for the occupants of specific environments. This may either be because the latter simply do not pay attention to the atmospheric/environmental cues (and hence do not register their incongruency) and/or because they have no reason to believe that the stimuli should be combined in the first place.

### Sensory dominance

One common feature of configurations of multisensory stimuli that are in some sense incongruent is sensory dominance. And very often, under laboratory conditions, this tends to be vision that dominates (e.g., Hutmacher, 2019; Meijer et al., 2019; Posner et al., 1976). Under conditions of multisensory conflict, the normally more reliable sense sometimes completely dominates the experience of the other senses, as when wine experts can be tricked into thinking that they are drinking red or rosé wine simply by adding some red food dye to white wine (Wang & Spence, 2019). Similarly, people’s assessment of building materials has also been shown to be dominated by the visual rather than by the feel (Wastiels, Schifferstein, Wouters, & Heylighen, 2013; see also Karana, 2010).

At the same time, however, while we are largely visually dominant, the other senses can also sometimes drive our behaviour. For instance, according to an article that appeared in the *Wall Street Journal*, many people will apparently refuse to check in to a hotel if there is funny smell in the lobby (Pacelle, 1992). Such admittedly anecdotal observations, were they to be backed up by robust empirical data, would then support the notion that olfactory atmospheric cues can, at least under certain conditions, also dominate in terms of determining our approach-avoidance behaviour. Meanwhile, a growing number of diners have also reported how they will sometimes leave a restaurant if the noise is too loud (see Spence, 2014, for a review; Wagner, 2018), resonating with the quote from Blesser and Salter (2007) that we came across a little earlier.

One other potentially important issue to bear in mind here concerns the “assumption of unity”, or coupling/binding priors that constitute an important factor modulating the extent of crossmodal binding in the case of multisensory object/event perception, according to the literature on the currently popular Bayesian causal inference (see Chen & Spence, 2017; Rohe, Ehlis, & Noppeney, 2019, for reviews). Coupling priors can be thought of as the internalized long-term statistics of the environment (e.g., Girshick, Landy, & Simoncelli, 2011). Does it, I wonder, make sense to suggest that we have such priors concerning the unification of environmental/atmospheric cues? Or might it be, perhaps, that in a context in which we are regularly exposed to incongruent environmental/atmospheric multisensory cues - just think of how music is played from loudspeakers without any associated visual referent - that out priors concerning whether to integrate what we see, hear, smell, and feel will necessarily be related, in any meaningful sense, may well be reduced substantially. See Badde, Navarro, and Landy (2020) and Gau and Noppeney (2016) on the role of context in the strength of the common-source priors multisensory binding.

Hence, no matter whether one wants to create a tranquil space (Pheasant, Horoshenkov, Watts, & Barret, 2008) or one that arouses (Mattila & Wirtz, 2001), the senses interact as they do in various other configurations and situations (e.g., Jahncke, Eriksson, & Naula, 2015; Jiang, Masullo, & Maffei, 2016). There are, in fact, numerous examples where the senses have been shown to interact in the experience and rating of urban environments (e.g., Ba & Kang, 2019; Van Renterghem & Botteldooren, 2016).

### Crossmodal correspondences in architectural design practice

**Fig. 10:**
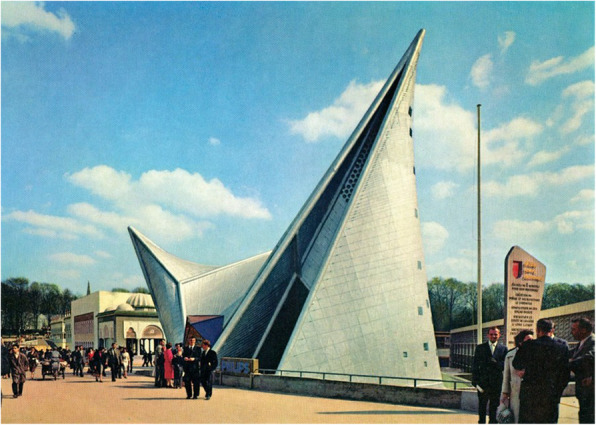
Philips pavilion was a World’s Fair pavilion designed for Expo 1958 in Brussels by the office of Le Corbusier. The building, which was commissioned by the electronics manufacturer Philips, was designed to house a multimedia spectacle of sound, light and projections celebrating post-war technological progress. Iannis Xenakis was responsible for much of the project management. [Figure copyright Wikimedia Commons: Wouter Hagens]

The field of synaesthetic design has grown rapidly in recent years (e.g., Haverkamp, 2014; Merter, 2017; Spence, 2012b). According to architectural historian, Alberto Pérez-Gómez, mentioned earlier, the Philips Pavilion designed by Le Corbusier for the 1958 Brussels world’s fair (Fig. 10) attempted to deliver a multisensory experience, or atmosphere by means of “forced” synaesthesia (Pérez-Gómez, 2016, p. 19).^18^ The interior audiovisual environment was mostly designed by Le Corbusier and Iannis Xenakis (see Sterken, 2007). From those descriptions that have survived there were many coloured lights and projections and a looping soundscape that was responsive to people’s movement through the space (Lootsma, 1998; Muecke & Zach, 2007).

True to his oculocentric approach, mentioned at the start of this piece, Le Corbusier apparently concentrated on the visual aspects of the “Poème Electronique”, the multimedia show that was projected inside the pavilion. Meanwhile, his site manager, Iannis Xenakis created “Concret PH” - the soundscape, broadcast over 300 loudspeakers, that accompanied it. It is, though, unclear how much connection there actually was between the auditory and visual components of this multimedia presentation. The notion of parallel, but unconnected, stimulation to eye and ear comes through in Xenakis’ quote that: “we are capable of speaking two languages at the same time. One is addressed to the eyes, the other to the ears.” (Varga, 1996, p. 114). Moreover, in his later work (e.g., Polytopes), Xenakis pursued the idea of creating a total dissociation between visual and aural perception in large abstract sound and light installations (Sterken, 2007, p. 33).

At several points throughout his book Pérez-Gómez (2016), stresses the importance of “synaesthesia” to architecture, without, unfortunately, ever really quite defining what he means by the term. All one finds are quotes such as the following: “primordial synesthetic perception*”*, p. 11; “perception is primordially synesthetic”, p. 20; “synaesthesia as the primary modality of human perception”, p. 71. Pérez-Gómez (2016, p. 149) draws heavily on Merleau-Ponty’s (1962, p. 235) *Phenomenology of Perception*, quoting lines such as: “The senses translate each other without any need of an interpreter, they are mutually comprehensible without the intervention of any idea.” A few pages later he cites Heidegger “truths as correspondence” (Pérez-Gómez, 2016, p. 162). This does, though, sound more like a description of the ubiquitous crossmodal correspondences (Marks, 1978; Spence, 2011) than necessarily fitting with contemporary definitions of synaesthesia, though the distinction between the two phenomena admittedly remains fiercely contested (e.g., Deroy & Spence, 2013; Sathian & Ramachandran, 2020). Abath (2017) has done a great job of highlighting the confusion linked to Merleau-Ponty’s incoherent use of the term synaesthesia, that has, in turn, gone on to “infect” the writings of other architectural theorists, such as Pérez-Gómez (2016).

Talking of synaesthetic design may then be something of a misnomer (Spence, 2015), the fundamental idea here is to base one’s design decisions on the sometimes surprising connections between the senses that we all share, such as, for example, between high-pitched sounds and small, light, fast-moving objects (e.g., Spence, 2011, 2012a). It is important to highlight the fact that while these crossmodal correspondences are often confused with synaesthesia, they actually constitute a superficially similar, but fundamentally quite different empirical phenomenon (see Deroy & Spence, 2013).

We have already come across a number of examples of crossmodal correspondences being incorporated, knowingly or otherwise, in design decisions. Just think about the use of temperature-hue correspondences (Tsushima et al., 2020; see Spence, 2020a, for a review). The lightness-elevation mapping (crossmodal correspondence) might also prove useful from a design perspective (Sunaga, Park, & Spence, 2016). And colour-taste and sound-taste correspondences have already been incorporated into the design of multisensory experiential spaces (e.g., Spence et al., 2014; see also Adams & Doucé, 2017; Adams & Vanrie, 2018). Once one accepts the importance of crossmodal correspondences to environmental design, then this represents an additional level at which sensory atmospheric cues may be judged as congruent (e.g., see Spence et al., 2014). One of the important questions that remains for future research, though, is to determine whether there may be a priority of one kind of crossmodal congruency over others when they are manipulated simultaneously.

## Conclusions

While it would seem unrealistic that the dominance, or hegemony (Levin, 1993), of the visual will be overturned any time soon, that does not mean that we should not do our best to challenge it. As critic David Michael Levin puts it: “I think it is appropriate to challenge the hegemony of vision – the ocular-centrism of our culture. And I think we need to examine very critically the character of vision that predominates today in our world. We urgently need a diagnosis of the psychosocial pathology of everyday seeing – and a critical understanding of ourselves as visionary beings.” (Levin, 1993, p. 205). While not specifically talking about architecture, what we can all do is to adopt a more multisensory perspective and be more sensitive to the way in which the senses interact, be it in architecture or in any other aspect of our everyday experiences.

By designing experiences that congruently engage more of the senses we may be better able to enhance the quality of life while at the same time also creating more immersive, engaging, and memorable multisensory experiences (Bloomer & Moore, 1977; Gallace & Spence, 2014; Garg, 2019; Spence, 2021; Ward, 2014). Stein and Meredith (1993, p. xi), two of the foremost multisensory neuroscientists of the last quarter century, summarized this idea when they suggesting in the preface to their influential volume *The merging of the senses* that: “The integration of inputs from different sensory modalities not only transforms some of their individual characteristics, but does so in ways that can enhance the quality of life. Integrated sensory inputs produce far richer experiences than would be predicted from their simple coexistence or the linear sum of their individual products.”

There is growing interest across many fields of endeavour in design that moves beyond this one dominant, or perhaps even overpowering, sense (Lupton & Lipps, 2018). The aim is increasingly to design for experience rather than merely for appearance. At the same time, however, it is also important to note that progress has been slow in translating the insights from the academic field of multisensory research to the world of architectural design practice, as noted by licensed architect Joy Monice Malnar when writing about her disappointment with the entries at the 2015 Chicago Architecture Biennial. There, she writes: “So, where are we? What is the current state of the art? Sadly, the current research on multisensory environments appearing in journals such as The Senses & Society does not appear to be impacting artists and architects participating in the Chicago Biennial. Nor are the discoveries in neuroscience offering new information about how the brain relates to the physical environment.” (Malnar, 2017, p. 153).^19^ At the same time, however, the adverts for at least one new residential development in Barcelona promising residents the benefits of “Sensory living” (*The New York Times International Edition* in 2019, August 31–September 1, p. 13), suggests that at least some architects/designers are starting to realize the benefits of engaging their clients’/customers’ senses. The advert promised that the newly purchased apartment would “provoke their senses”.

Ultimately, it is to be hoped that as the growing awareness of the multisensory nature of human perception continues to spread beyond the academic community, those working in the field of architectural design practice will increasingly start to incorporate the multisensory perspective into their work; and, by so doing, promote the development of buildings and urban spaces that do a better job of promoting our social, cognitive, and emotional well-being.

## Data Availability

Not applicable.

## Footnotes

1 It is, though, worth highlighting the fact that the denigration of the sense of smell in humans, something that is, for example, also found in older volumes on advertising (Lucas & Britt, 1950), turns out to be based on somewhat questionable foundations. For, as noted by McGann (2017) in the pages of *Science*, the downplaying of olfaction can actually be traced back to early French neuroanatomist Paul Broca wanting to make more space in the frontal parts of the brain (i.e., the frontal lobes) for free will in the 1880s. In order to do so, he apparently needed to reduce the size of the olfactory cortex accordingly.

2 Or, as Tuan (1977, p. 18) once put it: “an object or place achieves concrete reality when our experience of it is total, that is, through all the senses as well as with the active and reflective mind”

3 Relevant here, Mitchell (2005) has suggested that there are, in fact, no uniquely visual media.

4 This an issue close to my own heart currently, as the Department where I work was closed due to the discovery of large amounts of asbestos (see BBC News, 2017). The university and the latest firm of architects involved in the project are currently battling it out to determine how much of the new building will be given over to individual offices versus shared open-plan offices and hot-desking. The omens, I have to say (at least pre-pandemic), from what is happening elsewhere in the education sector, do not look good (Kinman & Garfield, 2015).

5 Here, one might also consider the Abercrombie & Fitch clothing brand. For a number of years, the chain also managed to craft a distinctive dance sound to match the dark nightclub-like appearance of their interiors.

6 Writer Tanizaki (2001), in his essay on aesthetics *In Praise of Shadows*, also draws attention to the close interplay that exists, or better said, once existed, between architectural design and food/plateware design in traditional Japanese culture.

7 Intriguingly, Kirshenblatt-Gimblett (1991, p. 416) describes the white cube as an apparatus for “single-sense epiphanies”.

8 This despite Baudelaire’s line that the smell of a room is “the soul of the apartment” (quoted in Corbin, 1986, p. 169).

9 It is also worth noting how suggestible people can be concerning the presence of an odour, as first demonstrated by Slosson’s (1899) classic classroom demonstration of students in the lecture theatre detecting a fictitious odour in the air.

10 It has also been suggested that the energy crisis in the 1970s may also have been partly to blame, as that tended to result in lower ventilation standards.

11 Indeed, one might wonder whether the latter quote refers more to oral stereoagnosis (Jacobs, Serhal, & van Steenberghe, 1998), than specifically to gustation (see also Waterman Jr., 1917, for the suggestion that the tongue can be more revealing than the hand).

12 This response is very different from the aesthetic disappointment, or even disgust, felt by the man once hypothetically described by the philosopher Immanuel Kant who was very much enjoying listening to a nightingale’s song until realizing that he was listening to a mechanical imitation instead (Kant, 2000).

13 The owner of the car park did not like the sound of this particular sonic intervention, meaning that the researchers were unable to try it out in the field.

14 At the same time, however, one might consider how marble, one of the most highly prized building materials is in some sense incongruent, given the rich textured patterning of the veined appearance of the surface is typically perfectly smooth to the touch.

15 These were the anchors on three of the bipolar semantic differential scales used in this study.

16 The value of connecting with nature in architectural design practice was stressed by an advertorial for an arctic hideaway that suggests that: *“True luxury today is connecting with nature and feeling that your senses work again”* as appeared in an article in Blue Wings magazine (December 2019, p. 38).

17 It should, though, be remembered, that sometimes incongruency may be precisely what is wanted. Just take the following quote regarding the crossmodal contrast of thermal heat combined with visual coolness from Japan as but one example: “In the summer the householder likes to hang a picture of a waterfall, a mountain stream, or similar view in the Tokonama and enjoy in its contemplation a feeling of coolness.” (Tetsuro, 1955, p. 16).

18 Though Pérez-Gómez (2016, p. 65) seems to be using a rather unconventional definition of synaesthesia, as a little later in his otherwise excellent work, he defines perceptual synaesthesia as “the integrated sensory modalities”, Pérez-Gómez (2016, p. 65). The majority of cognitive neuroscientists would, I presume, take this as a definition of multisensory perception, rather than synaesthesia. Synaesthesia, note, is typically defined as the automatic elicitation of an idiosyncratic concurrent, not normally experienced, in response to the presence of an inducing stimulus (Grossenbacher & Lovelace, 2001).

19 Eberhard (2007, p. xv) sounds a similarly pessimistic note writing that: “I doubt very much that neuroscientific findings will ever usurp intuition and inspiration as a guiding principle within architecture”.
